# Acute Headache at Emergency Department: Reversible Cerebral Vasoconstriction Syndrome Complicated by Subarachnoid Haemorrhage and Cerebral Infarction

**DOI:** 10.1155/2015/503871

**Published:** 2015-02-10

**Authors:** M. Yger, C. Zavanone, L. Abdennour, W. Koubaa, F. Clarençon, S. Dupont, Y. Samson

**Affiliations:** ^1^Unité Neurovasculaire, Hôpital de la Pitie-Salpetrière, APHP, 75013 Paris, France; ^2^Université Pierre et Marie Curie, 75006 Paris, France; ^3^Service de Rééducation Neurologique, Hôpital de la Pitie-Salpetrière, APHP, 75013 Paris, France; ^4^Unité de Réanimation Neurologique, Hôpital de la Pitie-Salpetrière, APHP, 75013 Paris, France; ^5^Neuroradiologie, Hôpital de la Pitie-Salpetrière, APHP, 75013 Paris, France; ^6^Service d'Épilpeptologie, Hôpital de la Pitie-Salpetrière, APHP, 75013 Paris, France

## Abstract

*Introduction.* Reversible cerebral vasoconstriction syndrome is becoming widely accepted as a rare cause of both ischemic and haemorrhagic stroke and should be evocated in case of thunderclap headaches associated with stroke. We present the case of a patient with ischemic stroke associated with cortical subarachnoid haemorrhage (cSAH) and reversible diffuse arteries narrowing, leading to the diagnosis of reversible vasoconstriction syndrome. *Case Report.* A 48-year-old woman came to the emergency department because of an unusual thunderclap headache. The computed tomography of the brain completed by CT-angiography was unremarkable. Eleven days later, she was readmitted because of a left hemianopsia. One day after her admission, she developed a sudden left hemiparesis. The brain MRI showed ischemic lesions in the right frontal and occipital lobe and diffuse cSAH. The angiography showed vasoconstriction of the right anterior cerebral artery and stenosis of both middle cerebral arteries. Nimodipine treatment was initiated and vasoconstriction completely regressed on day 16 after the first headache. *Conclusion.* Our case shows a severe reversible cerebral vasoconstriction syndrome where both haemorrhagic and ischemic complications were present at the same time. The history we reported shows that reversible cerebral vasoconstriction syndrome is still underrecognized, in particular in general emergency departments.

## 1. Introduction

Reversible cerebral vasoconstriction syndrome (RCVS) is characterised by acute and severe headaches, with or without other acute neurological symptoms, associated with diffuse segmental constrictions of cerebral arteries that resolve spontaneously within 3 months.

There is a clear female predominance of RCVS with sex ratio ranging from 2,6 : 1 to 10 : 1 [1, 2].

Three large series of RCVS in different countries have contributed to the consideration of this syndrome as a cause of stroke [1–3].

Because of reporting biases, the frequency of stroke in RCVS is uncertain, ranging from 3 [1] to 30% [3]. Haemorrhagic complications (intracerebral haemorrhage and cortical subarachnoid haemorrhage, cSAH) occur mainly in the week after the first headache, whereas ischemic events occur preferentially later (two weeks after the first headache). cSAH is also more frequent (12%) than ischemic events (6%) [4]. The physiopathology of RCVS seems to involve genetic features, endothelial dysfunction, and variation of the adrenergic cerebral tone [4]. This physiopathology is close to that of posterior reversible encephalopathy syndrome and, therefore, the two entities are assimilated by some authors. Since it remains poorly understood, the treatment of RCVS is uncertain and not consensual. We aim to expose, through a single but precise description of a case, our own experience of a complicated RCVS.

## 2. Case Report

**Figure 1:**
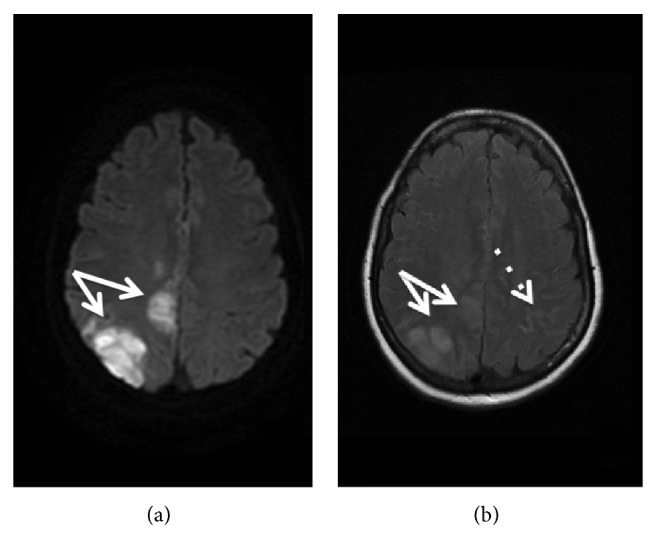
Diffusion-weighted imaging (a) and fluid-attenuated inversion recovery imaging (b) MRI of the patient, showing cytotoxic oedema in the right middle cerebral artery and anterior cerebral artery territories (plain arrows) and cSAH (discontinued arrow).

**Figure 2:**
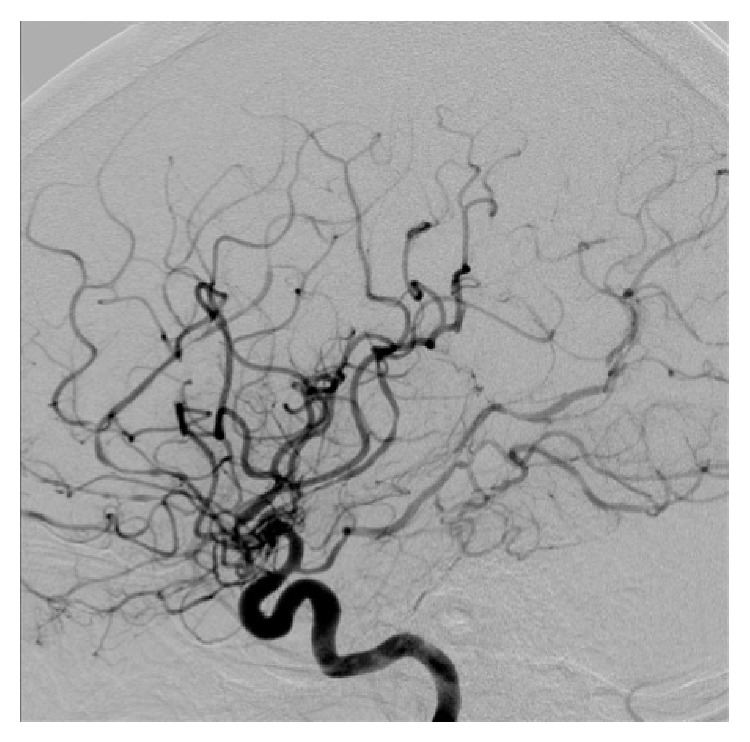
Digital subtraction angiography with injection of the right internal carotid artery showing narrowing of the arteries of both carotid and vertebrobasilar circulations.

A 48-year-old woman was admitted to the emergency department of a community hospital for an unusual thunderclap occipital headache associated with nausea, phonophobia, and photophobia, reaching peak intensity within one minute. Headache occurred during an emotional stress (while crying after having been informed of the death of a friend). She had never experienced such a brutal and painful headache. There was no prior history of headaches. Brain computed tomography (CT) and CT-angiography performed at the emergency department were unremarkable. On day four, the patient came back to the emergency department, because of the persistence of her headache. She was reassured and was rapidly discharged home without any further brain imaging. On day 11, she presented a sudden left hemianopsia and she was thus hospitalized. A brain magnetic resonance imaging (MRI) with diffusion-weighted imaging was performed the day of admission and showed hyperintense lesions in the right frontal lobe in the territory of anterior cerebral artery and in the right occipital lobe in the territory of posterior cerebral artery indicative of cytotoxic oedema. The same lesions were visible on fluid-attenuated inversion recovery imaging sequence. Images compatible with a cSAH were localised to the cortical sulcus in the right superior frontal lobe (Figure 1). Magnetic resonance angiography demonstrated diffuse severe arterial narrowing in the anterior and posterior circulations bilaterally. Injection of cervical vessels with gadolinium and fat saturation sequences excluded the presence of cervical dissections. On day 12, the patient experimented sudden weakness of the left upper and lower extremity and was immediately referred to our stroke unit. On admission, her blood pressure was 120/70 mmHg, her pulse 64 beats per minute, and her temperature 36°C. The neurologic examination showed left hemiparesis predominant to the lower extremity, dysarthria, and spatial neglect. The National Institute of Health Stroke Score was 11. A second MRI was performed and showed an extension of ischemic lesions in frontal and occipital lobes and diffuse cSAH. Magnetic resonance angiography showed poor vascularisation of bilateral anterior and posterior circulations. A CT did not show recent cSAH and CT-angiography image showed vasoconstriction of the right anterior cerebral artery and stenosis of bilateral middle cerebral arteries, as well as vasoconstriction of the bilateral posterior cerebral arteries and vertebral arteries. A bedside transcranial Doppler ultrasonography revealed bilateral and asymmetrical elevated mean middle cerebral arteries velocities (right middle cerebral artery: 240 cm/s; left middle cerebral artery: 150 cm/s) and elevated mean anterior cerebral arteries velocities (right anterior cerebral artery: 102 cm/s; left anterior cerebral artery: 190 cm/s). Oral nimodipine (120 mg/day) and intravenous hydration were initiated and the patient was referred to the resuscitation department. Digital subtraction angiography performed on day 13 showed typical segmental vasoconstriction in the anterior and posterior circulation (Figure 2). There was neither sinus thrombosis nor arteriovenous malformation. A control of the digital subtraction angiography on day 16 showed complete regression of vasoconstriction. A MRI performed on day 24 did not evidence new ischemic lesions and showed a regression of vasospasm. The patient had no neurological residual impairments.

We diagnosed her as having RCVS complicated by cSAH and ischemic lesions, on the basis of reversibility of the vasoconstriction and brain imaging findings.

## 3. Discussion

Acute severe headache presenting to the emergency departments accounts for 1-2% of admissions [5]. Even if RCVS is one of the diagnoses to be evocated in the setting of nontraumatic headache and neurological emergency [6], the history that we report here demonstrates that this condition is still underrecognized, as suggested by numerous authors [2]. Despite a clinical and radiological presentation compatible with RCVS, the patient has been discharged from the emergency department without any advice or medication. The precipitating factor (emotional stress due to the death of a friend) may have led the emergency physicians to the wrong conclusion but could be a real source of adrenalin surge that might be related. We can suppose that an earlier diagnosis may have prevented severe complications. In front of a clinical presentation of RCVS, after having ruled out other differential diagnoses, advice should be given to the patient such as resting, avoiding any vasoconstrictive medication, and coming back in case of reoccurrence of a thunderclap headache or apparition of a new symptom.

Nimodipine is commonly used in RCVS without any proof of its efficiency on the outcome. Resting can also improve the outcome of RCVS, since thunderclap headaches occur especially during physical activity.

Even if haemorrhagic complications occur preferentially sooner than ischemic ones [4] the patient experienced a severe RCVS with both haemorrhagic and ischemic complications at the same time (day 12). She had no prior history of headache but she was aged 48 years, an age more frequently linked with haemorrhagic complications [4]. This case reminds physicians to be careful with any clinical presumption of RCVS, especially in a woman in the forties-fifties.

## References

[1] Ducros A., Boukobza M., Porcher R., Sarov M., Valade D., Bousser M.-G. (2007). The clinical and radiological spectrum of reversible cerebral vasoconstriction syndrome. A prospective series of 67 patients. *Brain*.

[2] Chen S.-P., Fuh J.-L., Wang S.-J. (2010). Reversible cerebral vasoconstriction syndrome: an under-recognized clinical emergency. *Therapeutic Advances in Neurological Disorders*.

[3] Singhal A. B., Hajj-Ali R. A., Topcuoglu M. A. (2011). Reversible cerebral vasoconstriction syndromes: analysis of 139 cases. *Archives of Neurology*.

[4] Ducros A. (2012). Reversible cerebral vasoconstriction syndrome. *The Lancet Neurology*.

[5] Ward T. N., Levin M., Phillips J. M. (2001). Evaluation and management of headache in the emergency department. *The Medical Clinics of North America*.

[6] Schwedt T. J., Matharu M. S., Dodick D. W. (2006). Thunderclap headache. *The Lancet Neurology*.

